# Fu's subcutaneous needling for subcutaneous adhesions and scar hyperplasia in the neck region: A case report

**DOI:** 10.1097/MD.0000000000021103

**Published:** 2020-07-17

**Authors:** Huixia Huang, Jin Liu, Mingquan Fu, I-Wen Lin, Li-Wei Chou

**Affiliations:** a First Affiliated Hospital of Guangzhou University of Chinese Medicine; b Guangzhou University of Traditional Chinese Medicine, Guangzhou, China; c Chung Shan Medical University Hospital; d Department of Physical Medicine and Rehabilitation, China Medical University Hospital; e Department of Physical Therapy and Graduate Institute of Rehabilitation Science, China Medical University; f Department of Rehabilitation, Asia University Hospital, Taichung, Taiwan.

**Keywords:** Fu's subcutaneous needling, lymphadenectomy, postoperative adhesion, scar tissues, tongue cancer

## Abstract

**Rationale:**

Lymphadenectomy for tongue cancer in the neck region is often accompanied by local impaired mobility, gland damage, difficult in swallowing, and postoperative complication and seriously affects patients life quality. We reported a case of subcutaneous adhesions and scar hyperplasia in the neck region after lymphadenectomy for tongue lesions accompanied by impaired neck mobility and difficult in swallowing was treated using Fu's subcutaneous needling (FSN) treatment.

**Patient concerns:**

A 55-year-old male with tongue cancer received surgical intervention with lymphadenectomy 8 years ago was revealed a 15 cm-long curved surgical incision in the neck region and surrounded by numerous scar tissues.

**Diagnosis:**

Post-operation subcutaneous adhesions and scar hyperplasia in the neck region after lymphadenectomy was diagnosed.

**Interventions:**

FSN treatment was performed 2 to 3 times per week for 1 month to sway the affected tightened muscle and dissociate the superficial fascia beneath the scar resulted in a considerable improvement in neck movement.

**Outcomes:**

The Vancouver Scar Scale (VSS) was as follows: color (M) - 1; vascular distribution (V) - 0, thickness (H) - 2, and flexibility (P) - 4, with a total of 7 points before FSN treatment. The VSS after 1 month of FSN treatment was as follows: M1, V0, H2, and P2, with a total of 5 points. Neck mobility in different directions, i.e., stretching to the back of the neck and laterally bending the neck to the left and/or right side, was improved (*P* < .05).

**Lessons:**

At present, treatment of chronic scar hyperplasia has certain side effects and limitations. FSN is safe and convenient, with minimal destruction of the superficial fascia, having evident effects of dissociating tissue adhesion under scars and compensating for deficiencies in scar hyperplasia treatment. It can provide new ideas for future treatments.

## 1 Introduction

Tongue cancer, the most common malignant oral tumor today, often appears with local lymphatic metastasis, such as in the neck region.^[1]^ For patients with local or latent lymphatic metastasis, local lymphadenectomy or radiotherapy is satisfactory^[2]^; however, postoperative complications negatively affect a patients life quality. Fu's subcutaneous needling (FSN) acts on loose subcutaneous connective tissues and sways the surrounding fascia tissues. In combination with reperfusion approach, FSN plays a role in relaxing muscles and relieving symptoms. FSN is widely used to treat cervical spondylosis, peripheral facial paralysis, tension headache, and other muscular disorders.^[3–5]^

In our case report, a case of chronic subcutaneous adhesion and scar hyperplasia in the neck region after lymphadenectomy for tongue lesions at the First Affiliated Hospital of Guangzhou University of Chinese Medicine was treated with FSN, resulting in remarkable improvement in neck movement. The following case notes are provided to further study FSN and to improve patients life quality after the procedure.

## 2 Case presentation

**Figure 1:**
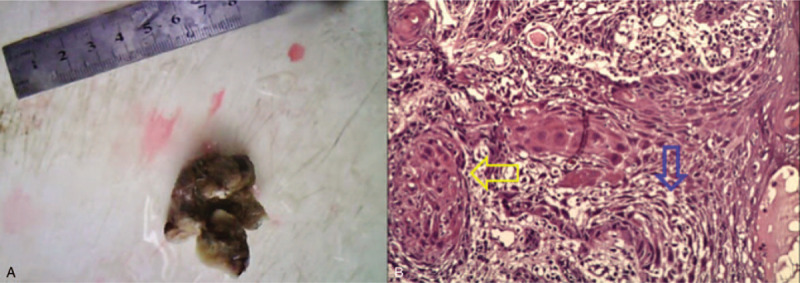
Tumors after left glotectomy and related pathogenesis (Note: A: Appearance of tumors and incisions [diameter of the removed tumor: 0.8 cm]; B: Squamous cell nests [indicated by yellow arrows] and keratin pearl [indicated by blue arrows] seen in the mass).

A 55-year-old male with impaired neck mobility and difficult in swallowing after tongue lesion resection with lymphadenectomy on June 23, 2011 was aware of paresthesia on the surface of his neck, and aggravated symptoms gradually. In February 2011, the patient was admitted to the First Affiliated Hospital of Guangzhou University of Chinese Medicine with recurrent oral ulcer. Physical examination revealed 2 ulcerated areas, 1 cm × 1 cm and 0.5 cm × 0.5 cm in size, on the left side of the tongue. The oral ulcer areas were biopsied and pathologically suspected as highly differentiated tongue squamous carcinoma. Postoperative pathology after left hemiglostectomy on February 19, 2011, showed (1) highly differentiated oral squamous cell carcinoma on the left tongue, with a mass diameter of approximately 0.8 cm and a depth of invasion of approximately 0.3 cm; and (2) leukoplakia on the left tongue without tumor invasion in the matrix or anterior, posterior, inner, or outer boundaries of the mass (Fig. 1).

Another mass was also found on the right side of the neck 4 months after surgery, and physical examination showed that there was no new mass noted in the residual tongue, but the lymph nodes under the right jaw were enlarged, with a size of approximately 2 cm × 2 cm and clear boundaries. Thus, cervical lymph node metastasis was considered. Pathological examination after neck lymphadenectomy on June 23, 2011, showed

**Figure 2:**
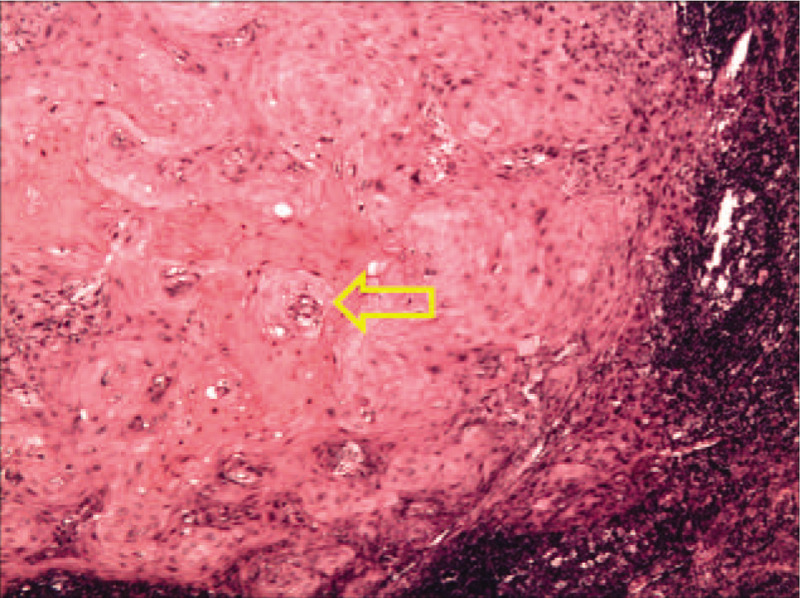
Pathology after cervical lymphadenectomy (Note: Atypical nest infiltrations [indicated by yellow arrows] composed of squamous cells arranged in layers).

1. highly differentiated metastatic squamous cell carcinoma in the right and left submaxillary lymph nodes;
2. reactive hyperplasia in the right submaxillary lymph nodes;
3. absence of cancer metastasis (0/8) in the left neck lymph nodes; and
4. cancer metastasis (2/4) in the right neck lymph nodes (Fig. 2).

**Figure 3:**
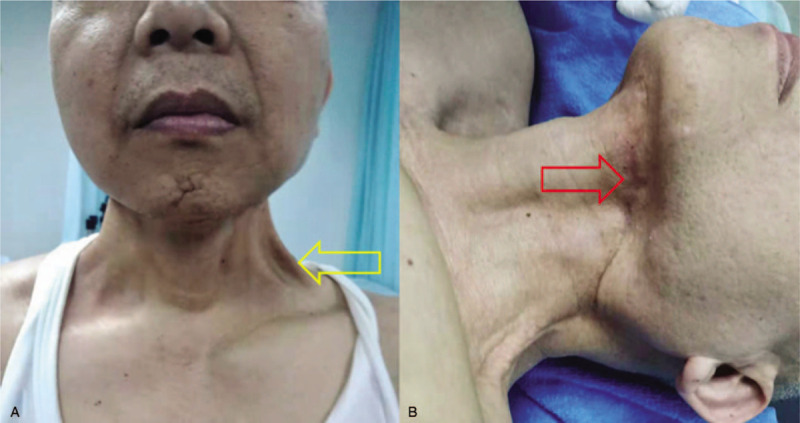
Appearance of muscles and surrounding scar tissues after surgery (Note: A: Changes in the appearance, shape, and location of muscles in the neck region [abnormal appearance and location of the sternocleidomastoid indicated by yellow arrows] and surrounding skin adhesions with a cord-like appearance. B: Underjaw with curved incision and numerous surrounding scar tissues [indicated by red arrows]).

The patient visited our characteristic Traditional Chinese Medicine (TCM) clinic in November 2018. Physical examination revealed a 15 cm-long curved surgical incision with numerous surrounded scar tissues in the neck region, and muscular dysplasia in the sternocleidomastoid muscle and the scalenus muscle (Fig. 3). The Vancouver Scar Scale (VSS) was as follows: color (M): 1; vascular distribution (V): 0, thickness (H): 2, and flexibility (P): 4, with a total of 7 points. The range of neck movement in all directions was as follows: flexion: 30.67°± 7.87°; extension: 38.83°± 7.25°; right lateral side-bending: 27.83°± 3.66°; left lateral side-bending: 26.00°± 2.97°; right rotation: 54.83°± 9.09° and left rotation: 53.67°± 10.82° (Table 1). These previous data indicated that the patients neck movement limitation and difficult in swallowing were related to the postoperative subcutaneous adhesion and scar hyperplasia in the neck region which induced localized circulation disturbance and limited range of motion of the neck. FSN treatment was performed to release the affected muscle and dissociate tissue adhesions.

**Table 1:**
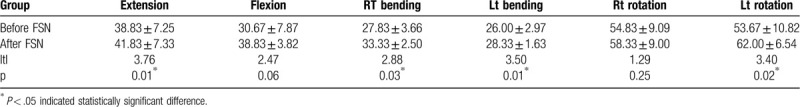
The range of neck movement in all directions before and after FSN treatment (X ± S°).

The patient relaxed his body naturally during the FSN treatment. The physician selected the muscle that felt cold, stiffness, numbness, or painful to the patient as the affected muscle, so called “tightened muscle (TM)”, including the bilateral sternocleidomastoid muscles, pectoralis major muscles, trapezius muscles, rectus abdominis muscles, diaphragm muscles, and erector spinae muscles. The entry point was located around the affected muscles, and needling was performed from the upper, lower, left, or right side or diagonally, whichever was the most convenient for the physician to operate. The needle was inserted into the muscle belly from far to near and mostly parallel or perpendicular to the muscle fibers. The main entry points were

1. sternocleidomastoid muscle: 3 to 5 cm above 1/3 of the clavicle or 3 cm below the Tiantu point (Ren 22);
2. trapezius muscle: at the Jianjing point (GB 21);
3. rectus abdominis muscle: 2 cm from the linea mediana ventralis; and
4. diaphragm muscle: 2 to 4 cm from the linea mediana ventralis and inside the cartilages of the 7th to 12th ribs.

The needle feeder for FSN was placed on the skin, and the angle between the needle, and the size of the entry point was maintained as small as possible. After the skin was disinfected with iodide, a disposable needle for FSN (Nanjing Paifu Medical Technology Co., Ltd.; Batch No. 20152270832) was inserted in parallel from the entry point into the loose subcutaneous connective tissues. If the physician obviously perceived resistance or the patient felt sore and swollen when the needle was inserted into the affected muscle, the needle was slightly pulled back to the subcutaneous layer.

The inserted needle was fixed to the skin with the thumb. The middle finger served as a support, and the index finger and the ring finger held the needle in turn to sway in a fan-shaped pattern at an angle of 40° with a frequency of 100 times per min for 2 minutes each, called “swaying movement. While the physician was swaying for FSN in tandem with reperfusion approach, the patient actively or passively contracted the affected muscle, forming a resistance to the physicians actions. The main reperfusion techniques were as follows.

**Figure 4:**
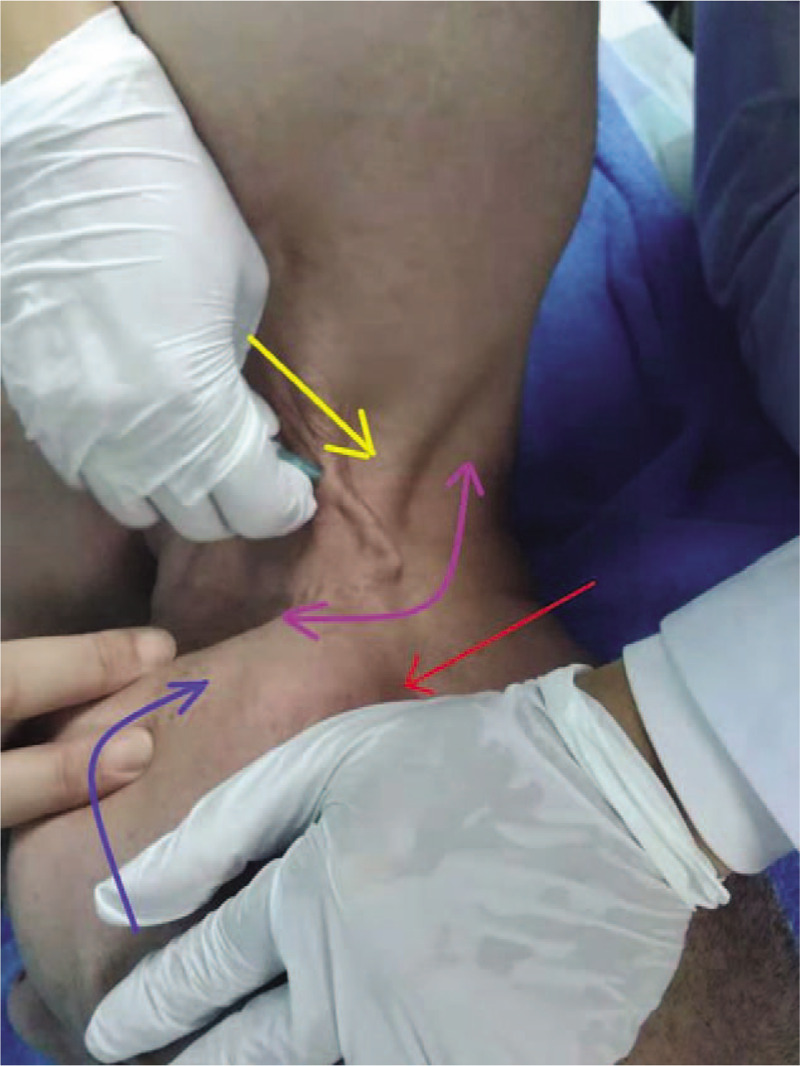
Reperfusion approach of the sternocleidomastoid muscle (Note: The sternocleidomastoid is the affected muscle, and needling direction is indicated by yellow arrows, with swaying at 40° in a fan-shaped pattern [indicated by purple arrows]; a physician presses the face [indicated by red arrows], and the patient turns his neck to the right to resist the resultant resistance [indicated by blue arrows]).

**Figure 5:**
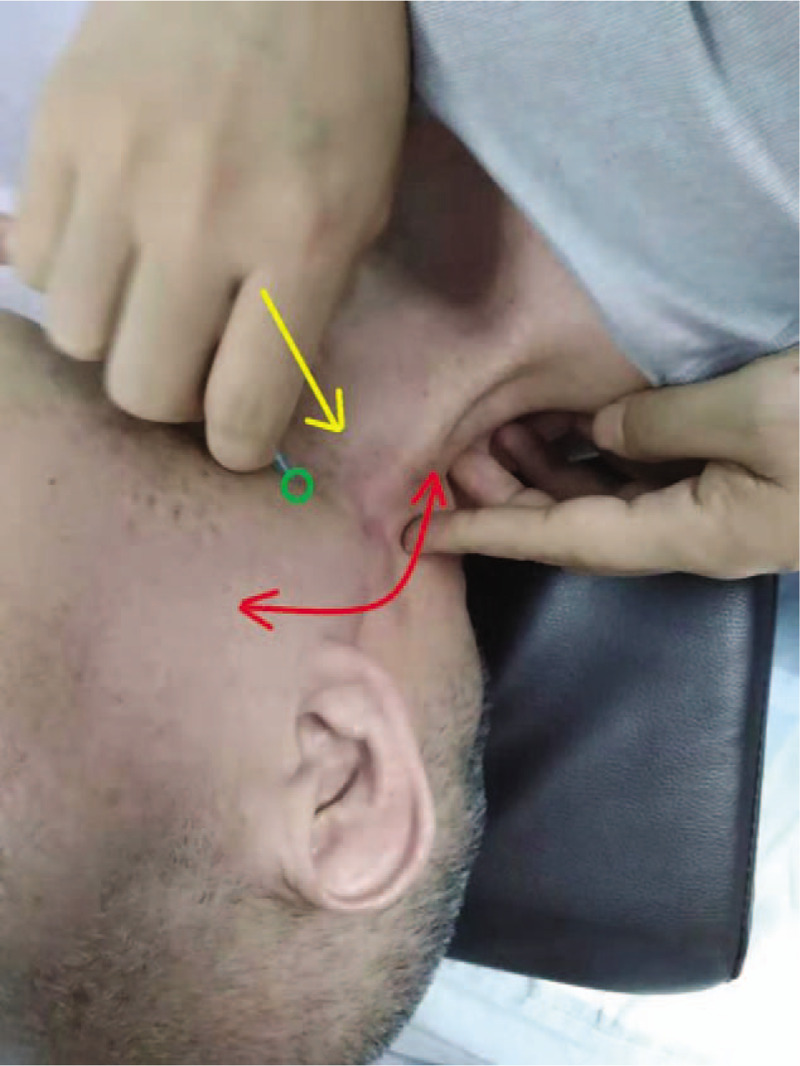
Swaying and dissociating connective tissues beneath scars (Note: After the needle was disinfected with iodide, the needle tip was inserted 2 cm from the scar tissue [indicated by green dots], with the needling direction parallel to the scar tissues [indicated by yellow arrows], swaying for 2 min beneath scars at the maximum angle [indicated by red arrows] with a frequency of 100 times per min).

1. For the sternocleidomastoid muscle, the patient lay down on a supine position, with the sternocleidomastoid fully exposed by turning his head to the normal side at 30° to 40°. With the entry point taken 3 cm above 1/3 of the clavicle, the needle tip was pushed toward the neck region, swaying in a fan-shaped pattern. The patients head was turned to the affected side, while the physician used his palm to hold the patients head and face and applied force to the opposite direction, forming an ipsilateral head-turning resistance (Fig. 4). Alternatively, the patient in the supine position had a fully exposed manubrium, and the entry point taken was 3 cm below the Tiantu point (Ren 22). The needle tip was pushed toward the neck region, swaying in a fan-shaped pattern. The patient might raise his head to the greatest extent, so the physician used his palm to hold the patients forehead and applied force to the opposite direction, forming a supine head-raising resistance.
2. For the trapezius muscle, the patient was in an orthopnea position, and the shoulder back on the affected side was fully exposed. The needle tip was pushed toward the neck region. The patient shrugged his shoulder on the affected side toward the same-side ear, and the physician held the shoulder on the affected side and applied force to the opposite direction, forming an ipsilateral shrugged shoulder resistance. Alternatively, the patient laterally bent his head toward the affected side, and the physician used his palm to hold the head and face on the affected side, forming an ipsilateral lateral bending head resistance.
3. For the rectus abdominis muscle, the patient was in a supine position, and his abdomen was fully exposed. The needle tip was pushed toward the linea mediana ventralis, and needling was performed in a direction perpendicular to the rectus abdominis muscle, thereby swaying in a fan-shaped pattern. The patient kept both lower extremities together, straight, and bent his body up at the hips with an angle of 30° or performed sit-ups with both upper limbs straightened and lifted off the bed at the same time.
4. For the diaphragm muscle, the patient was in a supine position with the chest and abdomen exposed and the needle tip pushed toward the xiphoid. As the patient took a deep breath and huffed, the physician used his palm to hold the patients abdomen and push.
5. For the cicatricose, the patient was in a supine position. For the physicians convenience, the entry point was taken 2 to 3 cm from the area (attachment point) where scar tissues were tightly connected, and the needling direction was parallel to the cicatricose. The needle tip was inserted into the connective tissues beneath the scars at the smallest possible angle, and the physician swept at the maximum angle and at the same frequency and duration as the affected muscle (Fig. 5), thereby forming an ipsilateral head-turning resistance and an ipsilateral lateral bending head resistance. Reperfusion approach lasted approximately 10 seconds each, with an interval of more than 10 seconds. The stiffness of the affected muscle was examined after reperfusion approach was repeated 3 times.

The patients condition should be carefully observed during FSN treatment, and the physicians approached technique, including swaying movement and reperfusion approach, should be smooth and soft. Upon the completion of the procedure, the physician should instruct the patient to move his body for a few minutes with the soft casing tube retained and observe improvement in the patients symptoms. If no dizziness, nausea, chest distress, palpitation, or any other adverse reaction was found, the disposable soft casing tube was removed.

FSN treatment was administered 2 to 3 times every week for 1 month until the patient felt a remarkable improvement in neck movement, and soreness, stiffness, cold, and tingling were greatly reduced. The VSS was M1, V0, H2, and P2, with a total of 5 points. The range of neck movement in all directions was as follows: flexion: 38.83°± 3.82°; extension: 41.83°± 7.33°; right lateral side-bending: 33.33°± 2.50°; left lateral side-bending: 28.33°± 1.63°; right rotation: 58.33°± 9.00° and left rotation: 62.00°± 6.54°. Stata11.0 was used to process data, and *t*-test for paired data was performed for statistical analysis, whilst *P* < .05 indicated statistically significant difference. A significant difference was observed in neck mobility in all directions except flexion and right rotation (*P* > .05) before and after treatment. Thus, neck movement was improved after the patient underwent FSN (Table 1).

## 3 Discussion

Tongue cancer is often accompanied by local lymphatic metastasis, mostly manifested as cervical lymph node metastasis.^[1]^ The incidence of cervical lymph nodes in tongue cancer is approximately 40% to 80%.^[6]^ Related investigations and statistical studies have shown that the prognosis of lymphatic metastasis is poor.^[7,8]^ Lymphadenectomy is the mainstream therapeutic regimen for patients with or at risk of lymphatic metastasis. Domestic studies have demonstrated that the resection and lymphadenectomy of primary lesions can significantly improve survival and prognosis. For patients at an early disease stage, lymphadenectomy can effectively reduce the tumor recurrence rate. Cheng et al^[9]^ conducted a series of follow-up studies on 146 patients with tongue cancer and found that those who receive primary tumor resection and lymphadenectomy at an early stage maintain 3- and 5-year recurrence rates of 7.9% and 16.9%, respectively, which are lower than those of patients who receive glossectomy (19.3% and 42.1%, respectively) (*P* < .05).

However, surgical treatment of tongue cancer is often accompanied by postoperative complications,^[10,11]^ including impaired mobility, logopathy, and ageusia.^[12–15]^ Lymphadenectomy severely damages the superficial fascia during the dissection of subcutaneous lymph nodes. Subcutaneous tissues are also called the superficial fascia. Loose connective tissues are the main constituents of the superficial fascia that contain numerous blood vessels, lymphatic vessels, nerves, and fibers inside and play a role in supporting, buffering, and nutrition. The sparse arrangement of loose connective tissues provides ample space for joint activities and buffer external stimuli.^[16]^ However, lymphadenectomy directly destroys the sparse arrangement of loose connective tissues, and postoperative inflammatory exudation, granulation tissue proliferation, and scar tissue formation further compress the space for subcutaneous tissues and limit the joint movements. In this case, the gap in subcutaneous tissues is narrowed. Adhesions and scar hyperplasia, accompanying neck movement limitation, difficult in swallowing, and other symptoms, occur. Paresthesia on the neck surface is related to the superficial nerve destruction inside the superficial fascia. Thus, releasing adhesions and dissociating tissue adhesions are prioritized during treatment.

FSN, an emerging acupuncture therapy innovated by Prof. Zhonghua Fu, improves the corresponding muscle–blood perfusion status by acting on the subcutaneous superficial fascia with swaying movement in the loose connective tissues, thereby alleviating muscle diseases.^[17]^ An affected muscle that is wholly or partially tighten in the normal motor center and in a state of natural relaxation is called the “tightened muscle”.^[18]^ Subcutaneous adhesions cause long-term tension on the muscles around the neck region, thereby blocking neck blood circulation. About the suspected mechanism of FSN therapy, loose subcutaneous connective tissues are thought as a crystal liquid environment that can effectively transfer energy, such as bioelectricity. In the swaying movement of FSN, the needle repeatedly sways the surrounding fascia tissues, thereby generating piezoelectric and reciprocal piezoelectric effects. Under such effects, cell ion channels are improved, and bioelectricity is diffused around and transmitted to distant muscles. Thus, the metabolism of tissue fluid and blood is improved, and neck pain or stiffness is alleviated.^[19,20]^ In combination with reperfusion approach, the pressure on local tissues of the affected muscle increases. When the tightened muscle is relaxed, peripheral blood perfusion increases as the perfusion range extends, and impairment to the neck mobility is further improved.^[21,22]^

Sternocleidomastoid muscle is one of the most superficial and largest cervical muscles, originates from the manubrium of the sternum and the clavicle to the mastoid process of the skull temporal bone, acting as the rotation of the head to the opposite side and flexion of the neck. The scar tissue was distributed over the bilateral sternocleidomastoid muscles and the mandibular area in this case, so the range of motion of neck was severe limited. We chose the sternocleidomastoid muscle as the majority of selected tightened muscle for FSN treatment. Neck movement limitation is also affected by the muscles of the chest, abdomen, waist, and back. The patients pectoralis major, diaphragm, rectus abdominis, and other muscles that are tighten and stiff are called tightened muscles. The muscles around the neck are chronically ischemic in the patient, and the movement of breathing or swallowing often requires the help of the muscles of the chest and abdomen in balancing the body cavity pressure. As a result, the pectoralis major, diaphragm, and other muscles are in a state of long-term tension compensation. Relaxing the tightened muscles of the chest, abdomen or waist may relieve neck stiffness to a certain extent.

In this case, dissociating connective tissues beneath scars, in addition to swaying the affected muscle and improving neck blood perfusion, is emphasized. Scar tissues are fibrous connective tissues formed after the maturation and aging of granulation tissue, thereby containing a high quantity of collagenous fibers that may shrink or adhere to the surrounding tissues and lead to joint contracture or limitation of motion.^[23]^ At present, the treatment of scar tissues mainly includes local injection, surface medication, physiotherapy, surgical operation, and photoelectron therapy, with certain limitations and side effects.^[24,25]^ FSN prioritizes swaying movement and is easily operated. The outer layer of the FSN needle is wrapped in a plastic tube. The swaying movement for FSN is equivalent to a “blunt dissection,” that is, minimal structural damage to blood vessels and lymphatics and high safety are observed. Swaying connective tissues beneath scars using FSN in tandem with reperfusion approach extends the sway range. Consequently, superficial fascia adhesions beneath the scar tissues are released, the space for subcutaneous tissues is widened, and a sufficient space for neck joints is cleared. As such, range of motion in neck is improved, and neck numbness and stiffness are relieved.

## 4 Conclusions

Lymphadenectomy for tongue lesion adversely damages the subcutaneous superficial fascia in the neck region. Postoperative adhesions and scar hyperplasia affect blood circulation and metabolism, then cause complications, such as impaired shoulder or neck mobility. FSN therapy has obvious effects on the treatment of muscle-related disorders and can effectively release loose subcutaneous connective tissues and dissociate tissue adhesions beneath scars. This procedure is safe and easily operated. It causes minimal damage to the body and can provide new ideas for future treatments.

## Acknowledgment

The authors thank Xinghua Chen and Lin Zhu for the conceptualization; and Zhonghua Fu for the technical consultation.

## Author contributions

**Methodology:** Huixia Huang, Jin Liu.

**Supervision:** Mingquan Fu.

**Writing – original draft:** Huixia Huang, Jin Liu, I-Wen Lin.

**Writing – review & editing:** Mingquan Fu, Li-Wei Chou.

## Correction

The article was originally published without the complete funding information and “The work was supported by China Medical University Hospital (CRS-108-047, DMR-109-094) and Asia University Hospital (10751003)" has been added to the footnote to correct this.
